# Load-induced inattentional deafness

**DOI:** 10.3758/s13414-014-0776-2

**Published:** 2014-10-07

**Authors:** Dana Raveh, Nilli Lavie

**Affiliations:** Institute of Cognitive Neuroscience, University College London, London, United Kingdom

**Keywords:** Divided attention, Inattention, Multisensory processing, Visual awareness, Perceptual load

## Abstract

High perceptual load in a task is known to reduce the visual perception of unattended items (e.g., Lavie, Beck, & Konstantinou, 2014). However, it remains an open question whether perceptual load in one modality (e.g., vision) can affect the detection of stimuli in another modality (e.g., hearing). We report four experiments that establish that high visual perceptual load leads to reduced detection sensitivity in hearing. Participants were requested to detect a tone that was presented during performance of a visual search task of either low or high perceptual load (varied through item similarity). The findings revealed that auditory detection sensitivity was consistently reduced with higher load, and that this effect persisted even when the auditory detection response was made first (before the search response) and when the auditory stimulus was highly expected (50 % present). These findings demonstrate a phenomenon of load-induced deafness and provide evidence for shared attentional capacity across vision and hearing.

Capacity limits on visual perception result in reduced visual detection ability in tasks involving high levels of visual perceptual load. Indeed, a range of inattentional blindness phenomena have been reported in task conditions of high perceptual load (e.g., Cartwright-Finch & Lavie, 2007; Jenkins, Lavie, & Driver, 2005 ; see Lavie, Beck, & Konstantinou, 2014 for a recent review; Lavie, Lin, Zokaei, & Thoma, 2009; Simons & Chabris, 1999). The original inattentional blindness paradigm (Mack & Rock, 1998) and many follow-up studies using it have measured subjective awareness reports (“noticed,” “did not notice”) with a surprise question at the very end of the experiment about an unexpected additional stimulus that was presented only once. Findings of inattentional blindness in studies using this procedure remain open to alternative accounts in terms of memory failures or intention rather than attention (Braun & Sagi, 1990; Wolfe, 1999). Importantly, inattentional blindness has also been demonstrated in conditions of high perceptual load in tasks that measure detection sensitivity for an expected stimulus that participants are instructed to detect (Carmel, Saker, Rees, & Lavie, 2007; Carmel, Thorne, Rees, & Lavie, 2011; Macdonald & Lavie, 2008). Together, all of these studies provide a convincing body of evidence for the effects of visual perceptual load on visual awareness; however, the cross-modal effects of perceptual load in vision on the awareness of an auditory stimulus remain as yet unclear. Understanding the effects of visual attention on hearing is important, both for a full multisensory model of attention and for daily-life applications. Although turning a “deaf ear” to the outside world when preoccupied with a visually loaded task may be advantageous at times (since it helps to focus attention on the task), there are of course situations in which this deafness is undesirable. For example, even in the most routine daily-life task of walking on the street, it is often vital that people not be deaf to the sound of people or vehicles approaching, despite the frequent engagement in various sources of visually loaded information (e.g., smartphones or shop windows). Previous studies on the effects of attention on auditory detection have typically varied the level of auditory perceptual load (e.g., Alain & Izenberg, 2003; Chait, Ruff, Griffiths, & McAlpine, 2012; Francis, 2010; Murphy, Fraenkel, & Dalton, 2013). However, the cross-modal effects of visual perceptual load on auditory perception have not been as widely explored, and the findings of the few existing studies have been mixed. A recent study by Parks, Hilimire, and Corballi (2011) examined the effect of visual perceptual load on neural responses to task-irrelevant auditory stimuli using steady-state evoked potentials. Participants monitored a central stream of crosses for targets defined on the basis of either a single feature (color) or a conjunction of features (color and orientation) and were simultaneously presented with irrelevant auditory distractors. Increasing visual load had no effect on potentials related to the unattended auditory stimuli. In contrast, in an earlier study Parks, Hilimire, and Corballis (2009) had used an almost identical manipulation of visual perceptual load and found that high visual perceptual load led to reduced amplitude of an auditory-evoked microreflex. This cross-modal effect is consistent with the results found by Macdonald and Lavie (2011), which provide preliminary behavioral evidence to suggest that a high level of visual perceptual load can result in inattentional deafness. In a series of experiments, participants made either low- or high-load visual discriminations, and on the last trial, a brief pure tone was presented simultaneously with the visual task display. The results showed that a greater number of participants failed to notice the presence of the auditory tone in the high-visual-load than in the low-load conditions; indeed, awareness rates in some cases dropped from 88 % in the low-load condition to 21 % in the high-load condition (Macdonald & Lavie, 2011, Exp. 2).

However, like results from the original inattentional blindness paradigm, these inattentional deafness reports may not necessarily reflect a true reduction in perceptual detection abilities. Because the auditory detection stimulus was only presented once per participant, detection sensitivity could not be assessed. The inattentional deafness reported may have therefore reflected, at least in part, the influence of a more stringent response criterion in the conditions of high perceptual load. Furthermore, because awareness of the auditory stimuli was assessed with a surprise question that followed the visual task response, effects of rapid forgetting during this delay may have been involved, too. Finally, Macdonald and Lavie’s (2011) findings are limited to reports about an unexpected stimulus. Many daily-life tasks involve the need to detect additional stimuli that are expected and that people are fully aware they should listen out for—for example, the sound of a timer going off for daily appliances, such as the oven.

In the present study, we sought to establish whether visual perceptual load can lead to reduced auditory detection sensitivity while addressing all these concerns. For that purpose, we modified Macdonald and Lavie’s (2011) inattentional deafness paradigm so that we could use a signal detection analysis with expected auditory stimuli that could also be responded to before the visual task response.

Participants performed a visual search task and were instructed to detect whether an auditory stimulus was present or absent during the search task performance. This presence-versus-absence detection task allowed us to assess signal detection sensitivity as well as response bias (Green & Swets, 1966). Finally, perception of the auditory detection stimulus was measured online rather than retrospectively, with response occurring straight after the visual task response, or even immediately upon presentation (Exp. 2).

## Experiment 1

In Experiment 1, participants were presented with a visual search task in which the level of perceptual load was manipulated through the similarity of the target and nontarget letters (e.g., Lavie & Cox, 1997). White noise was presented through the headphones during the presentation of each search display. Participants were asked to detect a pure tone (the auditory detection stimulus), which was occasionally (17 % of trials) presented amidst the white noise. Example trials with the auditory detection stimulus were presented at the start of the experiment.

### Method

#### Ethics statement

In this experiment, and all subsequent experiments reported here, participants were recruited from the UCL Psychology and Language Division (PALS) participant pool and gave their written consent to participate in the experiment. All the experiments reported were approved by the UCL PALS ethics committee.

#### Participants

Eighteen participants (ten women, eight men; mean age of 25.9 years, range of 19 to 45) participated in this experiment. Participants were excluded from this experiment, and from all subsequent experiments, if their accuracy on the letter search task was lower than 50 % and/or if their auditory detection accuracy rate was lower than 30 % in either, or both, of the load conditions. In this experiment, two participants were excluded and replaced because their accuracy on the letter search task was lower than 50 %. No participants were replaced due to low auditory detection rates. All of the participants in this experiment, as well as those in subsequent experiments, reported normal or corrected-to-normal vision and normal hearing and were able to detect the auditory stimulus used in the experiment upon hearing the sound examples. In addition, all were naive to the purposes of the experiments.

#### Apparatus and stimuli

**Fig. 1:**
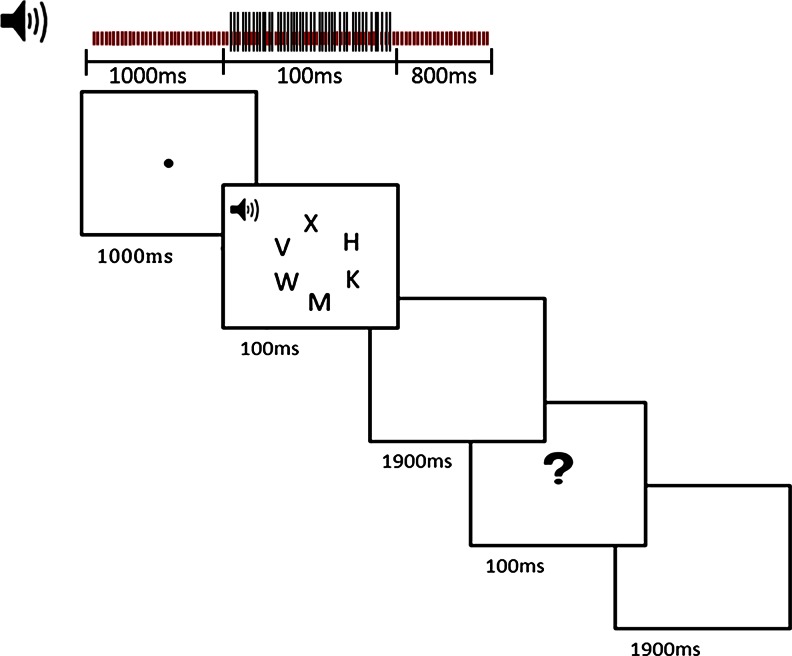
Example trial in Experiment 1, with an auditory tone present (17 % of all trials) in the high-load condition. In the low-load condition, the nontargets letters were all smaller *O*s. On the other 83 % of the trials, the tone was absent. In all trials (present and absent), a burst of white noise started with the onset of each trial’s fixation display and remained playing for 1.9 s. The tone was played during the 100-ms exposure of the visual task display. The search task response was made in a 2-s time window from the onset of the search display, and the auditory detection response was made during a 2-s time window from the onset of the question mark display

The experiments were created and run with E-Prime (Psychology Software Tools, Inc., 2003) on a Dell PC attached to a 13-in. monitor. A viewing distance of 57 cm was maintained with a chinrest throughout the experiment. All auditory stimuli were prepared with Wavosaur (version 1.0.5.0) and calibrated using a Brüel & Kjær type 2203 sound pressure meter. A pair of Philips SBC HP160 headphones were used to play the auditory stimuli to participants. On each trial, six equally spaced (nearest contours 1.1° apart) letters were presented in a circle (1.9° radius) that was centered at fixation (see Fig. 1 for a visual presentation of the letter search display). The background of the display was black and the letters were gray (red, green, blue [RGB] values: 180, 180, 180). The target letter, a capital letter *X* or *N* (0.6° × 0.6°, each equally likely), appeared at random but with equal probabilities at one of the six letter locations. The remaining five locations were occupied in the low-perceptual-load condition by smaller letter *O*s (0.2° × 0.2° wide) and in the high-perceptual-load condition by the letters *H*, *K*, *M*, *V*, *W*, and *Z* (of the same size as the target letter). White noise of 48 dB SPL was played continuously for 1.9 s during each trial, starting at the onset of the fixation display. On the critical trials, a 1025-Hz pure tone, at 28 dB SPL, was presented at the onset of the visual search display for 100 ms. The stimuli were presented in two blocks of 72 trials apiece, with the auditory detection stimulus being presented on 12 randomly selected trials per block (17 %). For each of the perceptual-load conditions, stimulus displays were counterbalanced with respect to target letter identity (*X* or *N*), target letter position (six possible letter positions), and the presence or absence of an auditory detection tone.

#### Procedure

At the start of each trial, a fixation dot was presented at the center of the screen for 1 s. This was then followed by the visual search task display for 100 ms (which included the auditory detection stimulus on 17 % of the trials), and subsequently a blank screen that lasted for 1.9 s. Therefore, in total, participants had 2 s during which they could make the search task response. Participants were instructed to make their search response as quickly and as accurately as possible. Next, a display with a question mark at the center was presented for 100 ms. Participants were asked to make the auditory detection response immediately upon the presentation of this question mark. This was followed by a blank screen for 1.9 s, thus giving participants a total of 2 s to make the auditory detection response (see Fig. 1 for a visual presentation of this design). To make the search task responses, participants were instructed to use their right hand to press the “0” key for the target *X* and the “2” key for the target *N* on the numeric key pad. For detection of the auditory stimulus, participants were instructed to press the “S” key with their left hand.

Before starting the main experiment, participants were shown three slowed-down example trials with the auditory detection stimulus (duration of 2 s instead of 100 ms for the visual search task display). This was followed by 12 normal-paced practice trials, with the auditory detection stimulus being presented on only two of the trials. Participants verbally confirmed whether or not they had heard the auditory detection stimulus, and for those who had failed to hear it at least three times, the example and practice trials were repeated. Each participant then completed two experimental blocks of 72 trials, both at the same level of perceptual load.

### Results and discussion

#### Letter search

Trials in which the search response was incorrect and those in which the reaction time (RT) was greater than 1.5 s were excluded from the search RT analysis in all of the experiments reported. One-way analyses of variance (ANOVAs) on the mean search RTs and error rates as a function of load revealed that search RTs were significantly longer in the high-perceptual-load condition (*M* = 935 ms, *SD* = 194.8) than in the low-perceptual-load condition (*M* = 706 ms, *SD* = 98.6), *F*(1, 16) = 9.88, *p* = .006, *η*_p_^2^ = .382, and search error rates were significantly higher in the high-perceptual-load condition (*M* = 19 %, *SD* = 9.1) than in the low-perceptual-load condition (*M* = 6 %, *SD* = 6), *F*(1, 16) = 12.79, *p* = .003, *η*_p_^2^ = .444. These results confirm that our manipulation of visual perceptual load was effective.

#### Auditory detection

Mean percentage correct detection rates, false alarm rates, *d'*, and response bias (*β*) were calculated and are shown in Table 1. Trials in which the search response was incorrect were excluded from the analysis (as in all of the subsequent detection analyses). One-way ANOVAs as a function of perceptual load indicated that correct detection rates were significantly lower in the high-perceptual-load condition than in the low-perceptual-load condition, *F*(1, 16) = 7.70, *p* = .014, *η*_p_^2^ = .325. The mean *d'* in the high-perceptual-load condition was also significantly lower than that in the low-perceptual-load condition, *F*(1, 16) = 11.58, *p* = .004, *η*_p_^2^ = .420. Values of *β* were not significantly different between the load conditions, *F*(1, 16) = 1.73, *p* = .207, *η*_p_^2^ = .098.

**Table 1:** Experiment 1: Mean percentage detection and false alarm rates and mean *d'* and *β* for the auditory detection stimulus as a function of perceptual load

Perceptual Load	Correct Detection Rate (%)	False Alarm Rate (%)	*d'*	*β*
Low	82	1.88	3.36	7.37
High	45	8.77	1.34	4.07

Since in the high-load condition the search task errors were higher than in the low-load condition, more critical trials were excluded from the analysis in the high-load condition (15 % excluded) than in the low-load condition (3 % excluded). However, even when the incorrect search task trials were included in the analysis, the correct-detection rate and *d'* were still significantly lower in the high-perceptual-load condition (correct detection rate, *M* = 44 %, *SD* = 35.1; *d'*, *M* = 1.31, *SD* = 1.7) than in the low-perceptual-load condition (correct detection rate, *M* = 81 %, *SD* = 21; *d'*, *M* = 3.31, *SD* = 0.85): *F*(1, 16) = 7.41, *p* = .015, *η*_p_^2^ = .316, for correct detection rates, and *F*(1, 16) = 10.18, *p* = .006, *η*_p_^2^ = .389, for *d'*. These findings provide preliminary evidence for the hypothesis that high visual perceptual load in a task reduces perceptual sensitivity in auditory detection.

## Experiment 2

Our hypothesis attributes the findings of reduced auditory detection sensitivity in conditions of high visual perceptual load to greater engagement of attentional capacity, and thus reduced capacity available for detection, as compared to the low-load conditions. However, because the detection response was made after the search task response in Experiment 1, an alternative account of the results in terms of memory failure remains plausible. Although, because of the fixed response window, the same time elapsed between the presentation of the auditory detection stimulus and the detection response in both load conditions, the longer RT in the search task under high load left less time for active maintenance of the auditory stimulus in the high-load condition. To address this concern in Experiment 2, we asked the participants to make their auditory detection response immediately upon the auditory tone presentation—that is, before the search task response, rather than after it, as in Experiment 1.

### Method

#### Participants

Eighteen new participants (11 women, seven men; mean age of 23.2 years, range of 18 to 30) took part. Three participants were replaced because their accuracy on the letter search task was lower than 50 %, and two were replaced because they detected less than 30 % of the beeps in either or both of the load conditions.

#### Stimuli and procedure

The apparatus, stimuli, and procedure were the same as in Experiment 1, except that the participants were instructed to make the detection response first, as soon as they heard the tone, and to respond to the letter search task afterward. If they did not hear the beep, they were instructed to respond to the letter search task as quickly and as accurately as they could. A single 2.7-s interval was available to make both responses.

### Results and discussion

#### Letter search

As in Experiment 1, longer search RTs and a greater number of errors were found in the high-perceptual-load condition (RTs, *M* = 1,086 ms, *SD* = 237.3; error rates, *M* = 29 %, *SD* = 7.3) than in the low-perceptual-load condition (RTs, *M* = 825 ms, *SD* = 235.5; error rates, *M* = 3 %, *SD* = 2.3): *F*(1, 16) = 5.27, *p* = .036, *η*_p_^2^ = .248, for the main effect of load on RTs, and *F*(1, 16) = 37.42, *p* < .001, *η*_p_^2^ = .701, for the load effect on error rates. These findings confirm that the manipulation of load was effective.

#### Auditory detection

Mean percentage correct detection rates, false alarm rates, *d'*, and *β* are presented in Table 2 as a function of perceptual load. Correct detection rates and *d'* were again significantly lower in the high-load than in the low-load condition: *F*(1, 16) = 6.84, *p* = .019, *η*_p_^2^ = .299, for correct detection rates, and *F*(1, 16) = 10.34, *p* = .005, *η*_p_^2^ = .393, for *d'.*

**Table 2:** Experiment 2: Mean percentage detection and false alarm rates and mean *d'* and *β* for the auditory detection stimulus as a function of perceptual load

Perceptual Load	Correct Detection Rate (%)	False Alarm Rate (%)	*d'*	*β*
Low	81	2.13	3.22	9.8
High	51	5.41	1.79	3.8

The results were unchanged when reanalyzed with the incorrect search trials included. Correct detection rates and *d'* were still significantly lower in the high-load condition (correct detection rate, *M* = 55 %, *SD* = 12.2; *d'*, *M* = 1.70, *SD* = 0.79) than in the low-load condition (correct detection rate, *M* = 81 %, *SD* = 11.8; *d'*, *M* = 3.21, *SD* = 0.78): *F*(1, 16) = 5.28, *p* = .035, *η*_p_^2^ = .248, for correct detection rates, and *F*(1, 16) = 11.18, *p* = .004, *η*_p_^2^ = .411, for *d'.*

As can be seen in Table 2, *β* was significantly lower in the high-perceptual-load than in the low-perceptual-load condition, *F*(1, 16) = 7.26, *p* = .016, *η*_p_^2^ = .312, indicating a more lenient criterion in the high-load condition. We will discuss the effect of perceptual load on response bias in further detail following Experiment 3, which we will see replicated this effect.

**Fig. 2:**
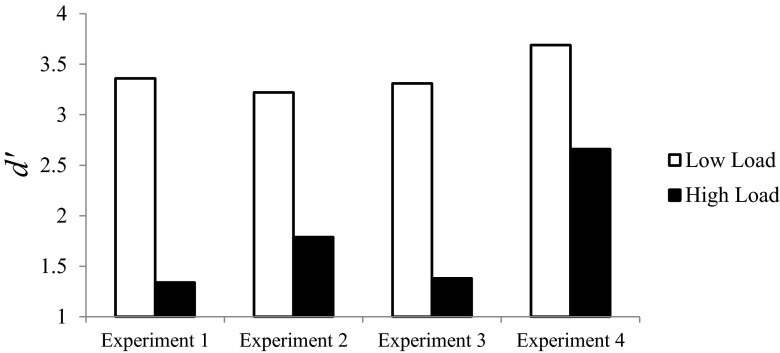
Auditory *d'* as a function of perceptual load in each experiment. Auditory detection sensitivity was consistently reduced with higher visual perceptual load in all four experiments. This effect persisted even when the auditory detection response was made straight upon the tone presentation (Exp. 2), as well as when the tone was highly expected and a detection response was required on every trial (Exp. 3). Furthermore, the effect generalized to presentations of a pure tone alone, with no background noise (Exp. 4)

Overall, Experiment 2 replicated the effect of visual perceptual load on auditory detection found in Experiment 1, even though the order of responses was reversed, so that the detection response came before the search response. Since this design allowed participants to immediately make their detection responses without delay, it allowed us to rule out alternative accounts of these effects in terms of memory rather than perception. A between-experiments ANOVA comparing the results of Experiments 1 and 2 (with Experiment and Load as between-subjects factors) further confirmed this, because no interaction effect on *d'* was found between perceptual load and experiment (*F* < 1); see Fig. 2.

## Experiment 3

So far, we have shown that visual perceptual load modulates auditory detection even in cases in which the auditory stimulus is expected. However, with the auditory stimulus only appearing on 17 % of the trials and participants only making detection responses if the auditory stimulus was present (and simply making no response if it was absent), it is plausible that the effects on perceptual sensitivity could be confined to cases in which both the expectancy of the auditory stimuli and the priority of the detection task were low. People may have simply neglected to attend and respond to the detection task on some occasions (a form of “goal neglect”; see, e.g., Duncan, Emslie, Williams, Johnson, & Freer, 1996), and this would be more likely to occur when the search task was more demanding, as in the conditions of high perceptual load. In other words, reduced priority for detection may account for the effects in addition to (or perhaps instead of) the reduced perceptual capacity in conditions of high load. To further test whether the effects of load on detection can be found in settings that discourage deprioritization of the detection task, in Experiment 3 we made the auditory detection stimulus highly expected, appearing on 50 % of trials, and participants’ detection responses (either present or absent) were now required on every trial, to prevent any potential effects of goal neglect.

### Method

#### Participants

Fourteen new participants (11 women, three men; mean age of 25.3 years, range of 18 to 45) took part. One participant was replaced because his accuracy on the letter search task was lower than 50 %, and one was replaced because he detected fewer than 30 % of the beeps in both of the perceptual load conditions.

#### Stimuli and procedure

The apparatus, stimuli, and procedure were the same as in Experiment 1, except that the beep was presented on 36 of the 72 trials (50 %) per block, and participants were instructed to press the “A” key when the beep was absent and the “S” key when the beep was present.

### Results and discussion

#### Letter search

As in Experiments 1 and 2, search RTs were significantly longer and error rates significantly higher in the high-perceptual-load condition (RTs, *M* = 1,087 ms; *SD* = 135; error rates, *M* = 18 %, *SD* = 12.7) than in the low-perceptual-load condition (RTs, *M* = 702 ms, *SD* = 217.7; error rates, *M* = 3 %, *SD* = 3.9)—*F*(1, 12) = 15.80, *p* = .002, *η*_p_^2^ = .568, for RTs, and *F*(1, 12) = 8.86, *p* = .012, *η*_p_^2^ = .425, for error rates—thus confirming that our manipulation of load was effective.

#### Auditory detection

The mean percentage correct detection rates, false alarm rates, *d'*, and *β* are presented as a function of perceptual load in Table 3. As can be seen in the table, the effects of perceptual load on correct detection rates and *d'* were replicated in Experiment 3: *F*(1, 12) = 5.88, *p* = .032, *η*_p_^2^ = .329, for correct detection rates, and *F*(1, 12) = 11.06, *p* = .006, *η*_p_^2^ = .48, for *d'.*

**Table 3:** Experiment 3: Mean percentage detection and false alarm rates and mean *d'* and *β* for the auditory detection stimulus as a function of perceptual load

Perceptual Load	Correct Detection Rate (%)	False Alarm Rate (%)	*d'*	*β*
Low	87	2.13	3.31	5.3
High	58	24	1.38	1.5

The results were unchanged when reanalyzed with the incorrect search trials included. Detection rates and *d'* were still significantly lower in the high-load condition (correct detection rate, *M* = 59 %, *SD* = 29.3; *d'*, *M* = 1.36, *SD* = 1.33) than in the low-load condition (correct detection rate, *M* = 86 %, *SD* = 8.92; *d'*, *M* = 3.29, *SD* = 0.58): *F*(1, 12) = 5.76, *p* = .034, *η*_p_^2^ = .324, for correct detection rates, and *F*(1, 12) = 12.42, *p* = .004, *η*_p_^2^ = .509, for *d'.*

Table 3 shows that *β* was lower in the high-load condition than in the low-load condition, *F*(1, 12) = 7.99, *p* = .015, *η*_p_^2^ = .400, demonstrating again a more lenient criterion in the high-load condition, as in Experiment 2, involving increased false alarm responses under high load (see Tables 2 and 3). This can be explained by pointing to the higher priority of the detection response in both of these experiments than in Experiment 1 (here because the auditory stimulus was highly expected and participants had to make a detection response on each trial, and in Exp. 2 because participants had to make their detection response first, before the search task response). The change in criterion is therefore likely to reflect a greater bias toward making detection responses when participants prioritized detection. Importantly, the effect of perceptual load on detection sensitivity was unaffected by the change in detection priority, as confirmed by the lack of interaction between load and experiment (*F* < 1) in an ANOVA comparing the effects of load between Experiments 3 and 1. Thus, high perceptual load reduced detection sensitivity despite the higher priority for detection, and the more lax response bias just led to increased false alarm rates under high load but did not improve detection accuracy. These results rule out an account of the effects of load on detection sensitivity in terms of deprioritization of auditory detection under high visual perceptual load.

## Experiment 4

Thus far, Experiments 1–3 have demonstrated that perceptual load in a visual search task modulates auditory detection of a pure tone embedded in white noise. In Experiment 4, we sought to examine whether this effect would also generalize across sound presentations that involved no noise, and so with a higher signal-to-noise ratio. For that purpose, in Experiment 4 we presented the same pure tone that had been used in Experiments 1–3, but alone, with no accompanying white noise.

### Method

#### Participants

Twenty new participants (ten women, ten men; mean age of 24.3 years, range of 18 to 43) took part. One participant was replaced because his accuracy on the letter search task was lower than 50 %. No participants were replaced due to low auditory detection rates.

#### Stimuli and procedure

The apparatus, stimuli, and procedure were the same as in Experiment 3, except that no white noise was played through the headphones on each trial, and on critical trials the same 1025-Hz pure tone at 28 dB SPL was presented at the onset of the visual search display for 100 ms.

### Results and discussion

#### Letter search

As before, search RTs were significantly longer and error rates were significantly higher in the high-perceptual-load condition (RTs, *M* = 928 ms, *SD* = 108.4; error rates, *M* = 27 %, *SD* = 10) than in the low-perceptual-load condition (RTs, *M* = 724 ms, *SD* = 173.4; error rates, *M* = 3 %, *SD* = 2.9)—*F*(1, 18) = 9.97, *p* = .005, *η*_p_^2^ = .356, for RTs, and *F*(1, 18) = 49.90, *p* < .0001, *η*_p_^2^ = .735, for error rates—thus confirming that our manipulation of load was effective.

#### Auditory detection

Mean percentage correct detection rates, false alarm rates, *d'*, and *β* are presented as a function of perceptual load in Table 4. The effects of perceptual load on correct detection rates and *d'* were replicated in Experiment 4: *F*(1, 18) = 4.79, *p* = .042, *η*_p_^2^ = .329, for correct detection rates, and *F*(1, 18) = 10, *p* = .005, *η*_p_^2^ = .48, for *d'.*

**Table 4:** Experiment 4: Mean percentage detection and false alarm rates and mean *d'* and *β* for the auditory detection stimulus as a function of perceptual load

Perceptual Load	Correct Detection Rate (%)	False Alarm Rate (%)	*d'*	*β*
Low	94	3	3.69	2.4
High	86	9	2.66	2

As in Experiments 1–3, the results were unchanged when reanalyzed with the incorrect search trials included. Correct detection rates and *d'* were still significantly lower in the high-load condition (correct detection rate, *M* = 86 %, *SD* = 8.2; *d'*, *M* = 2.71, *SD* = 0.76) than in the low-load condition (correct detection rate, *M* = 93 %, *SD* = 6.7; *d'*, *M* = 3.66, *SD* = 0.64): *F*(1, 18) = 4.87, *p* = .041, *η*_p_^2^ = .213, for correct detection rates, and *F*(1, 18) = 9.21, *p* = .007, *η*_p_^2^ = .339, for *d'.*

Although inspection of Fig. 2 suggests that the effect of perceptual load on sensitivity was smaller in this experiment than in Experiment 3, this difference was not significant [*F*(1, 30) = 1.92, *p* = .176, *η*_p_^2^ = .06, for the interaction of load and experiment in an ANOVA comparing the effects of load on *d'* between Exps. 3 and 4].

The values of *β* were not different between the low- and high-perceptual-load conditions in this experiment (*F* < 1). Since the absence of the white noise was the main difference between this experiment and Experiment 3, it is likely that the increased signal-to-noise ratio resulted in higher levels of confidence, leading to no adoption of a more lenient response strategy in the high-load conditions here.

#### Individual differences in capacity

Our claim of shared perceptual capacity between vision and hearing leads to the prediction that individuals with greater visual perceptual capacity should also have enhanced auditory detection ability. To examine this prediction, we conducted an individual differences analysis on the participants in all four experiments. A median split on the inverse efficiency scores (IEs = RTs/Accuracy rate) of visual search performance in the high-load condition allowed us to divide participants into low (IEs < 13.4) and high (IEs > 13.4) search capacity individuals. A one-way ANOVA revealed that mean *d'* was significantly higher in the high-span group than in the low-span group, *F*(1, 33) = 4.95, *p* = .034, *η*_p_^2^ = .142. This result provides further support for our claim that auditory detection sensitivity depends on the extent to which visual perceptual load resources are available.

#### Level of task training

Previous research had demonstrated that the effect of a demanding task on detection can be reduced with extensive training in either the specific task or similar tasks (e.g., Braun, 1998). Our task was not designed to examine the effects of training, and thus did not involve a large number of trials. Nevertheless, we could examine whether the level of more general training in other similar tasks had any impact, by comparing the effects between novice and more experienced participants (similarly to Braun’s, 1998, assessment of “task experience,” although without the same extensive level of training, involving thousands of trials in Braun, 1998). Of the 70 participants who took part in the present experiments, 19 had previously taken part in one or more visual attention experiments run in our laboratory, thus acquiring a higher level of general practice and experience with tasks of visual load involving brief stimulus presentations, such as those used in the present study. A two-way ANOVA conducted on auditory *d'*, with Perceptual Load (low, high) and the participants’ Prior Experience (novice, experienced) as between-subjects factors, revealed no main effect or interaction with experience (*F*s < 1 for both). In addition, a mixed ANOVA on auditory *d'*, with Perceptual Load as a between-subjects factor (low load, high load) and Block (1st, 2nd) as a within-subjects factor, revealed no significant main effect of specific task experience within our experiment, *F*(1, 68) = 1.59, *p* = .212, *η*_p_^2^ = .024, and no significant interaction between perceptual load and task experience (*F* < 1). These analyses suggest that the level of general experience or task-specific experience has no impact on the effect of visual perceptual load on auditory detection. It remains possible, however, that a more extensive as well as more specific training might produce an effect.^1^ This might be an interesting direction for future research.

## General discussion

The present research demonstrates that auditory detection sensitivity is consistently reduced with higher visual perceptual load in a letter search task, thus establishing a phenomenon of “load-induced deafness.” This phenomenon was found even when the auditory detection response was made straight upon the tone presentation (before the visual search response), as well as when the tone was highly expected (50 % present) and a detection response was required on every trial. Thus, alternative accounts of the findings in terms of memory, deprioritization, or goal neglect (cf. Duncan et al., 1996) were ruled out. Furthermore, the effect generalized to presentations of a pure tone alone, with no surrounding noise, and so to a higher signal-to-noise ratio than when stimuli were presented in the midst of white noise. This clarifies that the effects were not just due to a greater failure to separate signal from noise in conditions of high load (see, e.g., Stolte, Bahrami, & Lavie, 2014, for the effects of perceptual load on both signal gain and noise separation within vision). Further support for the claim that visual perceptual load critically determines auditory detection sensitivity comes from our finding that individuals with a larger visual perceptual capacity (as indexed by more efficient search performance) were also found to have enhanced auditory detection ability, relative to low-span individuals.

### Effects on sensitivity versus response criterion

We note that in two of our four experiments, visual perceptual load also led to a change in response criterion: High perceptual load led participants to use a more lenient response criterion, resulting in a greater rate of false alarms. Both of these experiments involved higher prioritization for the auditory detection task (with the auditory response being made first in Exp. 2 and with the auditory stimulus occurring on 50 % of trials in Exp. 3, in which participants had to respond “present” or “absent” on every trial). However, the lack of this effect in Experiment 4, which also involved higher prioritization, in the same manner as in Experiment 3, but no noise, suggests that the combination of high prioritization and the presentation of an auditory stimulus amidst noise (in Exps. 2–3) is what led to an effect of visual perceptual load on the response criterion.

Indeed, the combination of both high priority and added noise was unique in this set of experiments, as compared to previous research, and thus may explain the difference between the effects on criterion found for two of our experiments here and the consistent findings that within-modality effects of visual perceptual load on visual perception are found only for detection sensitivity, with no effects on criterion (e.g., Carmel et al., 2007; Carmel et al., 2011; Macdonald & Lavie, 2008). Importantly, given the independence of sensitivity and criterion measures in signal detection analysis, the effect of visual perceptual load on response criterion does not detract from our main findings concerning perceptual sensitivity.

### Shared perceptual capacity between vision and hearing

The present findings thus extend load theory to show that visual perceptual load is not only a critical factor for visual perception: The perceptual processes involved in detection sensitivity in hearing depend on the level of perceptual load in vision. This conclusion provides support for the load theory claim that, due to capacity limits in perception, perceptual processing is limited to just the most prioritized information in conditions of high perceptual load, and it generalizes this claim across the different modalities of vision and hearing. The cross-modal generalization of perceptual-load effects suggests a shared attentional capacity between vision and hearing, in line with a growing body of research suggesting the same, both in paradigms that have demonstrated the integration of visuo–audio information (e.g., Bertelson & Aschersleben, 1998; Driver, 1996; Shams, Kamitani, & Shimojo, 2000) and in those that have directly varied load in one modality and established modulations of processing in another modality (e.g., Berman & Colby, 2002; Brand-D’Abrescia & Lavie, 2008; Klemen, Büchel, & Rose, 2009; Parks et al., 2009). Our new findings clarify that the effects of shared capacity extend to perceptual sensitivity of auditory detection.

More specifically, however, our conclusion is in line with a few previous findings showing that engaging in tasks of high visual load can affect auditory processing, and it may provide an account for the previous findings in terms of reduced sensitivity for auditory perception with higher visual load. For example, Sinnett, Costa, and Soto-Faraco (2006) found that recognition rates for spoken words were reduced when participants had to also monitor a rapid stream of pictures, relative to when the words were fully attended. Santangelo, Olivetti Belardinelli, and Spence (2007) found that the effects of auditory spatial cueing (measured by the RT to a white noise presented on the same or the opposite side of a tone cue) were reduced when participants performed a demanding visual task (monitoring for digits among letters in a rapid stream), as compared to when participants were just performing the auditory cuing task. Macdonald and Lavie (2011) established that high (vs. low) visual load leads to inattentional deafness reports (i.e., greater rates of participants reporting that they did not notice an unexpected tone, as we described earlier, in the introduction). Although alternative accounts, in terms of a more strict response criterion or a lower weight for auditory input in conditions of dual tasks or of higher visual load, are possible for these findings, our study suggests that reduced perceptual sensitivity for hearing during high visual load can be the cause for all of these effects.

Our conclusion also provides an account for a striking demonstration that higher visual perceptual load (monitoring an RSVP stream for conjunctions of color and orientation, as compared to just color monitoring in the low-load condition) can reduce the amplitude of the postauricular reflex (the vestigial muscle response that pulls the ear backward) in response to a sudden auditory onset (Parks et al., 2009). Given the early nature of this reflex, its amplitude reduction with higher perceptual load is likely to result from reduced detection sensitivity to the tone itself (rather than from a direct modulation of the reflexive response under load).

Whilst the present study and previous other demonstrations of reduced auditory processing under high visual load point toward a shared capacity between vision and hearing, it is important to note that these demonstrations have all been obtained with assessments of the impact of visual load on auditory detection. The effects of auditory load may not always be as effective, for example, in a situation that was the reverse of the one tested here—namely, auditory load during visual perception. For instance, Rees, Frith, and Lavie (2001) found that the perception of visual motion is unaffected by higher auditory load, using a paradigm that they had shown modulates visual motion with higher visual load (Rees, Frith, & Lavie, 1997). Indeed, auditory load may also fail to have a within-modality effect on hearing (e.g., Chait et al., 2012 [violations of regularity]; Murphy et al., 2013). This may be attributed to the far lower spatial resolution of hearing than of vision, which results in auditory load not being as effective in keeping attention focused spatially, away from the detection stimulus. Thus, tests of shared capacity between vision and hearing may have better sensitivity with manipulations of visual load rather than of auditory load.

In addition, simultaneous, as opposed to successive, presentations of stimuli in both modalities may also play a role. Several studies using the “attentional blink” (AB) paradigm have reported that the reduction in perception of a second target when it is present within 300 ms of the first target in a successive presentation stream (i.e., the AB) is found only when the two targets are presented within the same modality, but not across the visual and auditory modalities (Duncan, Martens, & Ward, 1997; Hein, Parr, & Duncan, 2006; Potter, Chun, Banks, & Muckenhoupt, 1998; Soto-Faraco & Spence, 2002; Van der Burg, Olivers, Bronkhorst, Koelewijn, & Theeuwes, 2007). These findings could lead to the conclusion that when the auditory and visual stimuli are presented in a successive stream, and so are temporally separate (rather than integrated, as in our study), they do not share capacity. However, this conclusion has been contested through studies that have demonstrated a cross-modal AB, often using materials very similar to those used to demonstrate the absence of a cross-modal AB (Arnell & Jenkins, 2004; Arnell & Jolicœur, 1999; Arnell & Larson, 2002; Jolicœur, 1999; Ptito, Arnell, Jolicœur, & Macleod, 2008; Shulman & Hsieh, 1995; Soto-Faraco et al., 2002). Overall, the various conflicting demonstrations suggest that it is important to specify under what situations vision and hearing share or do not share perceptual capacity. For instance, the similarity between the second target (which is subject to the blink) and the distractors appears to play a critical role in the finding of a cross-modal AB (Arnell & Jenkins, 2004; see also Ptito et al., 2008, for a recent discussion of the role of task switching). Of course, different tasks may be sensitive to different situational factors; for example, it appears that in our task the failure to detect an auditory tone was found despite its being rather dissimilar from the letter items (both target and distractors) in the visual task.

Thus, our findings clearly provide evidence for the critical role of visual perceptual load in finding a cross-modal effect on detection in hearing (at least in so far as observers have not undergone earlier extensive specific training in the task). An interesting avenue for future research could be to delineate other critical factors.

### Comparing the effects of visual load on detection sensitivity in vision versus hearing

It is interesting to note that the effects of visual perceptual load on auditory detection reported here areequivalent in magnitude to those of visual perceptual load (using the same manipulation as here) on visual detection (Macdonald & Lavie, 2008). In our experiments, high load reduced auditory detection accuracy by 26 % and detection sensitivity by 1.6, on average, whereas Macdonald and Lavie (2008) found that the same manipulation of load reduced visual detection accuracy by 32 % and detection sensitivity by 1.5, on average (across their Exps. 1–5). Therefore, it appears that—at least for direct measures of detection under visual load—attentional capacity is shared between the modalities of vision and hearing.

### Applied implications

The present findings also have significant applied implications. The fact that people may be subjectively deaf to a sound when engaged with a task of high visual load has a range of consequences for daily life. For example, when engaged in a visually loaded task (e.g., searching for a missing jigsaw piece), people may fail to hear the doorbell ring, or may even fail to notice the sound of their car alarm from a distance. A pedestrian reading a text message when about to cross the road may fail to hear the sound of a vehicle approaching, and a surveillance operator may fail to hear a warning signal while monitoring a complex visual scene. The design of safety-critical operations and application should thus consider the level of visual load involved in the operator task.

## Footnotes

1 We thank Jochen Braun for suggesting this possibility.
